# Running with whole-body electromyostimulation improves physiological determinants of endurance performance – a randomized control trial

**DOI:** 10.1186/s13102-023-00739-8

**Published:** 2023-10-04

**Authors:** Anne Krause, Nicolas Walser, Christoph Centner, Daniel Memmert, Ivo da Mota de Moreia, Ramona Ritzmann

**Affiliations:** 1 Institute of Training and Computer Science, German Sport University Cologne, Cologne, Germany; 2 Praxisklinik Rennbahn, Muttenz, Switzerland; 3 Institute of Sport and Science, University of Freiburg, Freiburg, Germany

**Keywords:** Lactate, Metabolic demand, Endurance, Aerobic power, High-intensity training, Body composition

## Abstract

**Background:**

This study aimed to evaluate the physiological and metabolic adaptations to an eight-week running intervention with whole-body electromyostimulation (wbEMS) compared to running without wbEMS.

**Methods:**

In a randomized controlled trial (RCT), 59 healthy participants (32 female/ 27 male, 41 ± 7 years, rel.V̇O_2max_ 40.2 ± 7.4 ml/min/kg) ran twice weekly à 20 min for eight weeks either with a wbEMS suit (EG) or without wbEMS (control group, CG). Before and after the intervention, (i) rel.V̇O_2max_, heart rate and time to exhaustion were recorded with an incremental step test with an incremental rate of 1.20 km/h every 3 min. They were interpreted at aerobic and (indirect) anaerobic lactate thresholds as well as at maximum performance. (ii) Resting metabolic rate (RQ) as well as (iii) body composition (%fat) were assessed.

**Results:**

Following the intervention, V̇O_2max_ was significantly enhanced for both groups (EG ∆13 ± 3%, CG ∆9 ± 3%). Velocity was elevated at lactate thresholds and maximum running speed (EG ∆3 ± 1%, CG ∆2 ± 1%); HR_max_ was slightly reduced by -1 beat/min. No significant changes were observed for time until exhaustion and lactate. RQ was significantly enhanced following both trainings by + 7%. %fat was reduced for both groups (EG ∆-11 ± 3%, CG ∆-16 ± 5%), without any changes in body mass. Results did not differ significantly between groups.

**Conclusions:**

Both interventions had a positive impact on aerobic power. The rightward shift of the time-velocity graph points towards improved endurance performance. The effects of wbEMS are comparable to those after high-intensity training and might offer a time-efficient alternative to affect physiological and metabolic effects.

**Trial registration:**

German Clinical Trials Register, ID DRKS00026827, date 10/26/21.

**Supplementary Information:**

The online version contains supplementary material available at 10.1186/s13102-023-00739-8.

## Background

In nowadays society, the optimization of physical performance is becoming increasingly relevant. The primary objective is to reduce the time required for exercising while maximizing health effects and performance, such as after high-intensity training (HIT). HIIT is a training protocol alternating short periods of intense anaerobic exercise with brief recovery periods until the point of exhaustion. The efficiency of HIT on cardiovascular and metabolic function has widely been proven [1–3]. Despite its effectiveness, intensity and thus, the cardiovascular load of HIT is often perceived as too high. In the past decade, whole-body electromyostimulation (wbEMS) as an additional stimulus to voluntary muscle activation moved into focus as an alternative training method [4, 5].

WbEMS has primarily been researched in combination with strength training in rehabilitation to intensify the training or aid in the recovery process. Hereby, wbEMS was shown to improve strength modalities, such as power and maximal strength, as well as endurance performance [5–9]. Those adaptations were based on positive effects of wbEMS on the cardiorespiratory system and metabolism [5, 6, 10–12] including improved body composition [6, 13]. Effects were observed after just one training session [11, 12], ranging up to several weeks of training [6, 10, 14]. In contrast, only few studies have investigated the application of wbEMS to enhance endurance performance [6, 14, 15]. In a previous study with isolated EMS, Paillard and colleagues [4] found that EMS places a high demand on muscle metabolism and can increase energy consumption and carbohydrate oxidation more effectively than voluntary contraction. As described by Gregory and Bickel (2005), it is known that “electrical stimulation recruits motor units in a nonselective, spatially fixed, and temporally synchronous pattern” [16] which leads to a greater reliance on anaerobic glycolysis for energy production, resulting in the production of lactate and inorganic phosphate facilitating earlier fatigue [17, 18]. Since metabolic changes are crucial for muscle adaptation in endurance training [19], incorporating wbEMS alongside endurance exercise may potentially result in superior adaptations and performance improvements compared to traditional endurance training methods. In a recent experiment by our group, we demonstrated that wbEMS with running induced earlier fatigue and significant alterations in energy metabolism following a single exercise session [20].

The main objective of the current prospective, long-term trial was to examine the effects of repetitive training bouts for 8-weeks on endurance related performance parameters. Within the present randomized-controlled design, a new investigated the effects of a wire-free EMS suit (XENOMA), which allowed full range of motion and thus an optimal transfer to everyday activities or outdoor exercises. With the promising benefit of in-field-testing, we aimed to determine which metabolic and physiological effects are triggered throughout eight weeks interval running intervention with wbEMS as compared to without wbEMS with reference to endurance performance. We hypothesized that a training intervention with wbEMS leads to (i) an enhanced aerobic power and metabolic demand post-training, (ii) enhanced resting metabolic rate as well as (iii) improved body composition by means of body fat reduction.

## Materials and methods

### Experimental design

**Fig. 1:**
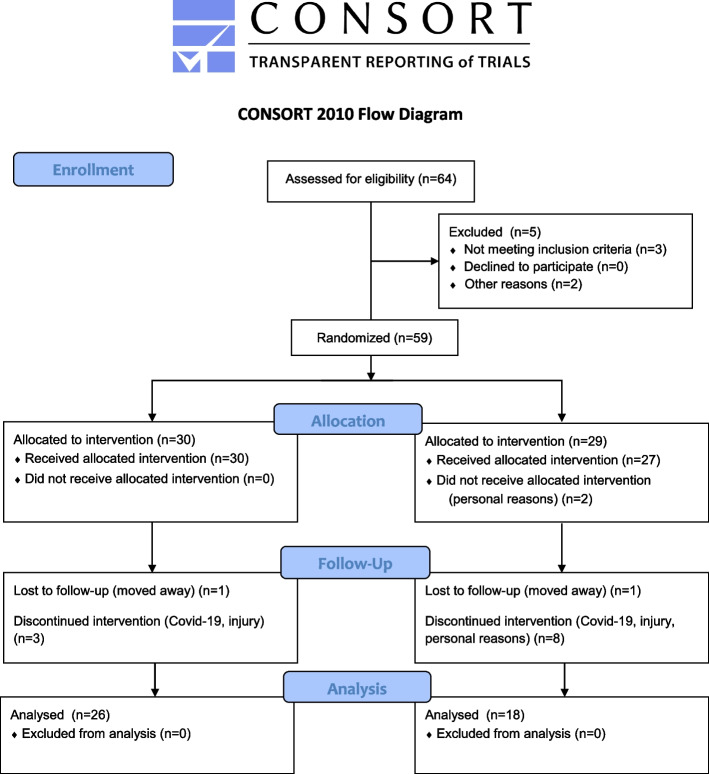
Flow chart of subject recruitment before and during the trial

To investigate potential long-term effects of superimposed wbEMS during running, a parallel two-group randomized controlled design was implemented. Before the start of the eight-week intervention, subjects were randomly and concealed allocated into one of the following groups (Fig. 1): running without (control group, CG) or running with additional wbEMS (EMS group, EG). All trainings and assessments were conducted at Biomechanics Praxisklinik Rennbahn (Switzerland), while data analysis was evaluated at the German Sport University Cologne (Germany). For allocation sequence generation, a random number generator was used.

In accordance with the latest revision of the Declaration of Helsinki, all subjects gave written informed consent to the experimental procedure which was approved by the ethics committee of the German Sports University (001/2021). The study is registered in the German Clinical Trials Register with the ID DRKS00026827. Experimental procedures and potential risks were explained, and informed consent was obtained before inclusion.

### Participants

Based on an a priori power analysis (G*Power V 3.1.9.2, mixed ANOVA, within-between interaction), a sample size of *n* = 52 volunteers was identified as sufficient to identify previously observed effect sizes as statistically significant (effect size = 0.2, power = 0.80, alpha = 0.05). Inclusion criteria were an age between 30 and 50 years, no or minor whole-body EMS (≤ once) and jogging-experience (< 4 km/ week). Exclusion criteria were pregnancy, neuronal, motor or metabolic diseases and orthopedic injuries, cardiovascular or respiratory diseases, dermatological illnesses, physical disability limiting the participants’ mobility and medication which affects physical performance. Before commencing training, all subjects attended a preliminary screening, which included a clinical anamnesis and physical examination to monitor agreement with the inclusion criteria. Participants were informed about contraindications [7, 21].

A total number of 59 healthy volunteers (32 female / 27 male, age 41 ± 7 years, body mass 73.7 ± 13.7 kg, height 173.6 ± 9.7 cm; rel. V̇O_2max_ 40.2 ± 7.4 ml/min/kg corresponds to performance level 1 according to De Pauw et al. [22] was recruited for the current study (Flow chart in Fig. 1). Thirteen participants (EMS: 4, noEMS: 9) dropped-out during the intervention due to the following reasons: Covid-19 (5), injuries (5) and personal reasons (3). Exclusion criteria were cardiovascular or respiratory diseases, dermatological illnesses, neurological diseases, physical disability limiting the participants’ mobility and medication which affects physical performance. Recruitment period lasted for three months 2021. There were no significant differences between groups (Table 1).

**Table 1:** Baseline anthropometric characteristics (mean ± standard error with confidence intervals 95%; participants that completed the trial)

	**EG**	**CG**	***p***
age [years]	40.4 ± 1.4 [37.6; 43.2] range 30–51	41.3 ± 1.8 [37.9; 44.8] range 30–53	2.77
height [cm]	175.6 ± 2.1 [171.6; 179.6] range 156–205	172.7 ± 1.8 [169.2; 176.2] range 162–184	0.57
Body mass [kg]	76.8 ± 2.8 [71.4; 82.2] range 51–113	69.3 ± 2.7 [64.0; 74.7] range 55–90	0.06
rel.V̇O2max [ml/min/kg]	40.9 ± 1.7 [37.5; 44.3] range 22–61	41.1 ± 0.9 [39.2; 42.9] range 32–47	1.40

### Training

Both groups exercised for eight weeks on two days of the week, with at least 48 h of rest between two sessions. Training duration was limited to 20 min [21]. During the first two weeks the first session was performed at the aerobic threshold (LT1). Every fortnight for the next four weeks 5 min at the (indirect) anerobic threshold (LT2) were added while time at LT1 was reduced by the same amount. The second session remained the same for the first 4 weeks and consisted of 2 × 4-min work intervals at max sustainable intensity (rating of perceived exertion > 7 out of 10) divided by 2-min recovery. For the remaining four weeks one more 4-min work interval was added. This interval training session was performed twice a week for the last 2 weeks of the intervention. Training intensities were derived of the incremental step test conducted before the training period.

For wbEMS (Xenoma, Japan), the procedure is described in Krause et al. [20]. Stimulation parameters were based on current scientific recommendations to ensure participant’s safety at all times in absence of muscle spasms or obstruction during running [14, 21, 23, 24]: Impulse type bipolar, frequency 85 Hz, width 180µs, rise 700 ms, duty cycle 50% (3 s on- and 3 s off-time). For each participant, the current was determined individually at a subjective tolerance rating of 7/10 with the CR10-scale [7] for every training session. Twenty-six electrodes transmitted the electrical current to muscles of the chest (4), abdomen (4), back (4), arms (4), hip (2), and thigh (8).

Training and suit stimulus intensity were controlled throughout the training process by trained personnel and are reported in Table 2.

**Table 2:** Intervention protocol

	**Week 1&2**		**Week 3&4**		**Week 5&6**		**Week 7&8**	
	Session 1	Session 2	Session 1	Session 2	Session 1	Session 2	Session 1	Session 2
**Total duration [min]**	20’	20’	20’	20’	20’	20’	20’	20’
**Protocol**	20’ vLT1	2 × 4’ max 2’ rest	15’ vLT1 5’ vLT2	2 × 4’ max 2’ rest	10’ vLT1 10’ vLT2	3 × 4’ max 2’ rest	3 × 4’ max 2’ rest	3 × 4’ max 2’ rest
**Frequency [Hz]**	85	85	85	85	85	85	85	85
**Duty cycle [%]**	50% (3:3)	50% (3:3)	50% (3:3)	50% (3:3)	50% (3:3)	50% (3:3)	50% (3:3)	50% (3:3)
**Median CR10 stimulus intensity**	6.3	7.0	7.0	7.0	7.0	7.0	7.0	7.0
**Median CR10 training intensity all (EG / CG)**	3 (4 / 2)	8 (8 / 8)	6 (6 / 4)	8 (8 / 8.5)	6.5 (7 / 6)	8 (8/ 8.3)	8 (8 / 8)	8 (8 / 8)

*EG* group with wbEMS suit, *CG* group without wbEMS suit, *vLT1* speed at aerobic lactate threshold, *vLT2* speed at anaerobic lactate threshold, *max* maximum intensity

### Assessments and outcome measures

Before and after the training period, assessment methods were conducted by a trained researcher to establish adaptations in performance, physiology and body composition.

As confounding variables, the dietary intake as well as the energy turnover were controlled. For the dietary intake, twenty representative participants reported their carbohydrate, fat, protein and total intake over a three-day period. The survey was conducted at the beginning and at the end of the intervention. For the energy turnover, everyday life activities in one week were recorded. The survey was conducted at the beginning and end of the training intervention.

#### Endurance performance and physiology

Breath-by-breath, respiratory data including oxygen uptake (V̇O_2_) were collected with a spirograph (Metalizer 3B, Cortex Biophysik GmbH, Leipzig, Germany; calibration 15% O_2_, 5% O_2_ BAL. N2). For each participant, starting velocity was individualized and set between 5.4 and 7.8 km/h [25]. Every 3 min, velocity was increased by + 1.2 km/ h until total exhaustion. Heart rate was monitored every 5 s during the step test to control for cardiac strain (Polar S710, PolarElectro GmbH, Büttelborn, Germany). Time and velocity were recorded until total exhaustion.

At baseline and after each step, 20 μl blood samples were taken from the ear lobe to determine blood lactate concentration (Lac). Samples were analyzed with Biosen C-Line Clinic / GP + (EKF-diagnostic GmbH, Barleben/ Magdeburg, Germany).

Aerobic (LT1) and (indirect) anaerobic thresholds (LT2) were determined with a detailed algorithm with the software ERGONIZER 4; for LT2, the modified Dmax method was used (Roecker et al. 2021). To compare physiological and metabolic values between trials, predefined points such as LT1, LT2 and maximum were chosen. All respiratory and HR data were averaged every 30 s.

#### Resting metabolic rate

Resting metabolic rate was calculated using indirect calorimetry. Subjects were instructed to arrive fasted without any physical activity prior to the test. All tests were conducted between 8 and 10am. Respiratory gas analysis was conducted using a spirograph (Metalizer 3B, Cortex Biophysik GmbH, Leipzig, Germany). Before each test, calibrations were performed on the flow meter with the use of a 3.0-L syringe and on the gas analyzers by using verified gases of known concentrations. Subjects rested quietly in the supine position in an isolated room with the temperature controlled to 21° C. RMR was measured for 20 min. Criteria for a valid RMR was a minimum of 5 min of steady state, determined as a < 10% fluctuation in oxygen consumption, < 6% fluctuation in carbon dioxide production and < 5% fluctuation in respiratory quotient. Oxygen consumption and carbon dioxide production were used to calculate RMR based on the Harris-Benedict equation [26].

#### Body composition 

Body composition by means of percentage body fat was assessed with a skinfold caliper (Holtain Ltd., Crymych, UK; precision = 0.2 mm) at seven anatomical sites (triceps, subscapular, iliac crest, supraspinal, pectoral, abdominal and thigh). Measurements were taken on the right side of body in absence of muscle contraction; the caliber was perpendicular to the site analyzed. Body fat was calculated according to the formula by Jackson, A.S. & Pollock, M.L. (1978, men) and Jackson and colleagues (1980, women).

### Statistics

For each dependent variable, a linear mixed model ANOVA (within-subject factor: time [2] x between-subject factor: group [2]) was calculated with the lmerTest package [27]. Level of significance was set at *p* < 0.05. Outliers, normality (Shapiro–Wilk test), homogeneity of variances (Levene test), assumption of sphericity (Mauchly’s test of sphericity) and homogeneity of covariances (Box’s m) were checked. Post-hoc tests were calculated with the Tukey correction for pair-wise comparison. Effect sizes are presented as generalized ${\eta }^{2}$ with reference values as follows: 0.01 = small, 0.06 = medium, 0.14 = large effect sizes [28]. Evaluation of baseline anthropometric characteristics was conducted with Student’s T-Tests and corrected according to Benjamini & Yekutieli [29].

All statistical analyses were performed with the statistical software R version 1.4.1717 and are presented as mean ± standard deviations, standard errors and with 95% confidence intervals.

## Results

Baseline values between EG and CG did not differ significantly. There were no significant changes in everyday life nutrition nor activities among the entire assessment period.

### Endurance performance and physiology

Time until total exhaustion did not change following both interventions with F(1, 43.69) = 4.04, *p* = 0.051.

V̇O_2max_ relative to body mass was enhanced after wbEMS by + 13% and after control by + 9% with F(1, 45.13) = 22.854, *p* < 0.001. Post-Hoc tests revealed a significant increase over time t(1, 45.30) = -4.48, *p* < 0.001, but not between groups at baseline (t(1, 69.40) = 0.907, *p* = 0.367) or after the intervention (t(1, 82.60) = 0.93, *p* = 0.355, Table 3).

**Table 3:** Descriptive and inference data of all dependent variables (mean ± standard deviation)

	**EG**		**CG**		**Anova**		**Effect sizes**	**Post hoc**
**parameter**	**pre**	**post**	**pre**	**post**	**F-values**	***p*-values**	${{\varvec{\eta}}}^{2}$**[95% CI]**	***p*-values**
**%Fat**	**21.0 ± 7.3**	**19.5 ± 6.9**	**21.5 ± 5.5**	**17.0 ± 4.6**	**F**_**time**_**(1, 43.02) = 20.77** F_group_(1, 55.03) = 0.11 F_interaction_(1, 43.02) = 3.95	< 0.001*** 0.74 0.05	**0.33 [0.15; 1.00]** 0.00 [0.00; 1.00] 0.08 [0.00; 1.00]	**p**_**time**_ < 0.001*** p_pre_ = 0.77 p_post_ = 0.37
**RQ**	**0.88 ± 0.1**	**0.92 ± 0.1**	**0.86 ± 0.1**	**0.91 ± 0.1**	**F**_**time**_**(1, 31.21) = 7.29** F_group_(1, 37.33) = 0.63 F_interaction_(1, 31.21) = 0.17	**0.01** 0.43 0.68	**0.19 [0.03; 1.00]** 0.02 [0.00; 1.00] 0.01 [0.00; 1.00]	**p**_**time**_** = 0.01** p_pre_ = 0.34 p_post_ = 0.73
Time_max_ [min]	24.8 ± 5.5	25.7 ± 6.2	23.2 ± 3.9	25.0 ± 4.3	F_time_(1, 43.69) = 4.04 F_group_(1, 55.43) = 1.53 F_interaction_(1, 43.69) = 0.03	0.05 0.22 0.86	0.08 [0.00; 1.00] 0.03 [0.00; 1.00] 0.00 [0.00; 1.00]	
**rel.V̇O**_**2max**_** [ml/min/kg]**	**41.0 ± 8.8**	**45.3 ± 9.0**	**39.2 ± 5.4**	**44.7 ± 6.4**	**F**_**time**_**(1, 45.13) = 22.85** F_group_(1, 55.73) = 1.03 F_interaction_(1, 45.13) = 0.01	< 0.001*** 0.32 0.91	**0.34 [0.16; 1.00]** 0.02 [0.00; 1.00] 0.00 [0.00; 1.00]	**p**_**time**_ < 0.001*** p_pre_ = 0.37 p_post_ = 0.35
Lac _LT1_ [mmol/ l]	1.59 ± 0.5	1.56 ± 0.6	1.65 ± 0.5	1.75 ± 0.6	F_time_(1, 48.12) = 0.02 F_group_(1, 55.95) = 0.61 F_interaction_(1, 48.12) = 0.34	0.89 0.44 0.56	0.00 [0.00; 1.00] 0.01 [0.00; 1.00] 0.01 [0.00; 1.00]	
**V**_**LT1**_** [km/h]**	**7.82 ± 1.6**	**8.25 ± 2.0**	**7.81 ± 1.4**	**8.78 ± 1.1**	**F**_**time**_**(1, 46.50) = 7.52** F_group_(1, 55.37) = 0.21 F_interaction_(1, 46.50) = 0.67	0.01** 0.65 0.42	**0.14 [0.02; 1.00]** 0.00 [0.00; 1.00] 0.01 [0.00; 1.00]	**p**_**time**_ = 0.01** p_pre_ = 0.99 p_post_ = 0.45
HR_LT1_ [1/min]	136 ± 15.6	133 ± 19.5	140 ± 16.8	144 ± 16.1	F_time_(1, 43.98) = 0.27 F_group_(1, 54.06) = 2.01 F_interaction_(1, 43.98) = 1.08	0.61 0.16 0.30	0.01 [0.02; 1.00] 0.04 [0.00; 1.00] 0.02 [0.00; 1.00]	
Lac _LT2_ [mmol/ l]	3.84 ± 1.2	3.84 ± 1.0	3.53 ± 0.9	3.61 ± 1.0	F_time_(1, 46,92) = 0.01 F_group_(1, 56.91) = 1.29 F_interaction_(1, 46.92) = 0.01	0.91 0.26 0.93	0.00 [0.00; 1.00] 0.02 [0.00; 1.00] 0.00 [0.00; 1.00]	
**V**_**LT2**_** [km/h]**	**11.08 ± 2.0**	**11.44 ± 2.2**	**10.57 ± 1.4**	**11.41 ± 1.2**	**F**_**time**_**(1, 42.44) = 23.13** F_group_(1, 55.13) = 1.01 F_interaction_(1, 42.44) = 0.12	< 0.001*** 0.32 0.73	**0.35 [0.17; 1.00]** 0.02 [0.00; 1.00] 0.00 [0.00; 1.00]	**p**_**time**_ < 0.001*** p_pre_ = 0.29 p_post_ = 0.37
HR_LT2_ [1/min]	170 ± 9.4	168 ± 13.4	170 ± 12.1	172 ± 12.3	F_time_(1, 43.80) = 1.65 F_group_(1, 55.63) = 0.27 F_interaction_(1, 43.80) = 1.28	0.21 0.60 0.26	0.04 [0.00; 1.00] 0.00 [0.00; 1.00] 0.03 [0.00; 1.00]	
Lac_max_ [mmol/ l]	8.85 ± 2.3	9.46 ± 2.5	8.34 ± 2.2	8.13 ± 2.0	F_time_(1, 46.08) = 0.11 F_group_(1, 56.32) = 2.96 F_interaction_(1, 46.08) = 2.71	0.74 0.09 0.11	0.00 [0.00; 1.00] 0.05 [0.00; 1.00] 0.06 [0.00; 1.00]	
**v**_**max**_** [km/h]**	**14.16 ± 2.5**	**14.45 ± 2.5**	**13.44 ± 1.7**	**14.25 ± 1.4**	**F**_**time**_**(1, 42.53) = 8.13** F_group_(1, 55.14) = 1.58 F_interaction_(1, 42.53) = 0.00	0.01** 0.21 0.95	**0.16 [0.03; 1.00]** 0.03 [0.00; 1.00] 0.00 [0.00; 1.00]	**p**_**time**_ = 0.01** p_pre_ = 0.22 p_post_ = 0.23
**HR**_**max**_** [1/min]**	**186 ± 8.9**	**185 ± 8.3**	**188 ± 11.3**	**187 ± 12.3**	**F**_**time**_**(1, 43.07) = 4.44** F_group_(1, 54.84) = 0.16 F_interaction_(1, 43.07) = 0.71	0.04* 0.69 0.40	**0.09 [0.00; 1.00]** 0.00 [0.00; 1.00] 0.02 [0.00; 1.00]	**p**_**time**_ = 0.04* p_pre_ = 0.50 p_post_ = 0.94

*EG* group with wbEMS suit, *CG* group without wbEMS suit, *RQ* resting metabolic rate, *HR* heart rate, *v* velocity, *Lac* lactate, *LT1* aerobic lactate threshold, *LT2* (indirect) anaerobic lactate threshold, *max* maximum

Heart rate did not change significantly over time at reference points HR_LT1_ (F(1, 43.98) = 0.27, *p* = 0.607) and HR_LT2_ (F(1, 43.80) = 1.65, *p* = 0.206). At maximum, a significant decrease was measured over time (F(1, 43.07) = 4.44, *p* = 0.041) with t(1, 43.90) = 2.10, *p* = 0.0412.

Lactate did not change significantly over time at any reference point such as for Lac_LT1_ (F(1, 48.12) = 0.02, *p* = 0.893), Lac_LT2_ (F(1, 46.92) = 0.01, *p* = 0.908) and Lac_max_ (F(1, 46.08) = 0.11, *p* = 0.741).

**Fig. 2:**
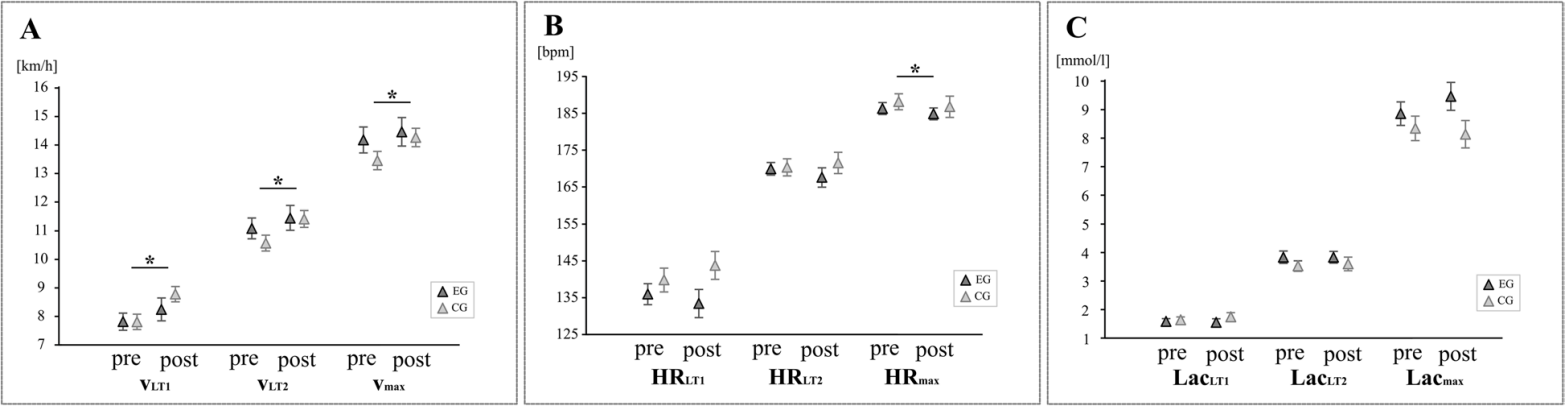
Mean values and standard errors before (pre) and after the intervention (post) of velocity (**A**), heart rate (**B**) and lactate (**C**) at reference points aerobic lactate threshold (LT1, triangles), (indirect) anaerobic threshold (LT2, rectangle) and maximum (max, circle)

Velocity increased at all reference points over time for both groups for v_LT1_ F(1, 46.50) = 7.516 (EG + 6%, CG + 11%, *p* = 0.009), for v_LT2_ F(1, 42.44) = 23.130 (EG + 4%, CG + 4%, *p* < 0.001) and for v_max_ with F(1, 42.53) = 8.132 (EG + 3%, CG + 2%, *p* = 0.007). Mean values are illustrated in Fig. 2. Post-Hoc tests were not significant between groups, but revealed a significant increase over time with v_LT1_ (1, 46.90) = -2.733, *p* = 0.009, v_LT2_ (t(1, 42.60) = -4.807, *p* < 0.001) and v_max_ (t(1, 42.70) = -2.85, *p* = 0.007, Table 3).

### Resting metabolic rate 

RQ increased significantly over time (F(1, 31.21) = 7.29, *p* = 0.011) with t(1, 46.80) = -2.69, *p* = 0.010.

### Body composition

Fat in relation to body mass decreased significantly for both groups over time (F(1, 43.02) = 20.771, *p* < 0.001). Body mass did not change significantly over time neither for EG (+ 0.02 ± 1.8 kg, *p* = 1.916) nor for CG (+ 0.6 ± 2.3 kg, *p* = 0.270, Table 1).

## Discussion

The aim of this study was to elucidate if running with wbEMS affects metabolic and physiological responses with reference to endurance performance. After eight weeks of running with wbEMS, (i) aerobic power (V̇O_2max_) and velocity at all reference points were enhanced. (ii) While RQ was slightly increased, (iii) relative body fat was reduced. No significant differences were observed between CG and EG which indicates that running with wbEMS has no superimposed impact as compared to running without wbEMS.

Outcomes of the experiment point towards equivalent effects of running for eight weeks with and without wbEMS on endurance performance. These effects include maximal oxygen uptake and its health-related impact, enhanced aerobic power and changes in body composition for both types of interventions.

First, maximal oxygen uptake, as a determinant of running performance [30], was significantly enhanced following both trainings. This is in line with previous investigations following six [6] and twelve weeks of wbEMS training [14] and reinforces the potential of running with and without wbEMS to improve aerobic power. Enhanced oxygen uptake is due to greater oxygen delivery and/or utilization (greater capillarization, mitochondrial density) and is comparable to adaptations following high-intensity training [31]. This is not only important for exercise tolerance but also for long-term health at mid and advanced ages [32–34]. Thereby, higher values of oxygen uptake are correlated with lower risk of cardiovascular events associated with declined risk of mortality [35].

Second, those parallels of improved aerobic power between wbEMS and HIT were also evident in regard to running speed. Velocity at LT-reference points is often used as a workload parameter [36]. Following eight weeks of running with wbEMS, speed was increased by + 6% at LT1, by + 4% at LT2 and by + 3% at maximum. Peak heart rate was slightly reduced by 1 beat/min which was probably due to heart rate variability; therefore it can be assumed that this had no effect on current results [37]. Termination speed, however, is known as being a reliable and valid parameter of endurance performance [38]. The current values are lower compared to previous investigations, which demonstrated an increase by + 20.5% at LT following 12 weeks of HIT [31] or by + 8–9% at ventilatory thresholds following six weeks of wbEMS training [6]. Enhanced running speed at predefined reference points is, in reverse, equivalent to reduced lactate at the same speed and can be interpreted as a rightward shift of the lactate curve in a velocity–time graph (see Fig. 2). This is as another indicator of improved endurance performance for interventions running with and without wbEMS [39].

Third, body composition was significantly affected following both interventions with a strong interaction tendency with *p* = 0.05. Skinfolds measures allow a reliable determination of subcutaneous body fat at seven skinfold sites [40]. Outcomes show that body fat decreased after the wbEMS intervention while RQ increased slightly by 7% for each group. While resting metabolic rate is one major determinant of the magnitude of fat-free mass, those results are quite surprising. Kemmler and colleagues observed a similar outcome in postmenopausal women following 14 weeks of adjuvant wbEMS strength training: no changes occurred in resting metabolic rate, but skinfold was significantly reduced [13]. It is noteworthy to discuss the changes in body composition with slight changes in RQ. As has already been shown in other studies [41], RQ at rest is strongly dependent on a variety of factors, such as muscle fiber composition and glycogen content as well as dietary fat intake. Although diet and activity were recorded in protocols, the influence of these factors cannot be finally excluded. Due to superimposed wbEMS training to running, energy consumption may be enhanced and thus a negative energy balance can be considered a physiological consequence. Additionally, and as described before, improved oxygen uptake points towards changes in capillary and mitochondrial density (greater oxygen uptake). It is known that those changes lead to higher lipid and lower glycogen depletion [31, 42] which is in line with our current results of reduced body fat.

### Limitations

For a conclusive statement, it is crucial to consider the limitations of the study. Two aspect are of substantial importance. 1. Although stratified for gender, age and running experience, the current pool of participants was quite heterogeneous with high variations among primary outcome parameters, which might have had a great impact on the current results. 2. The additional stimulus during running did not have an additional effect on the results as referring by no group differences. It can be assumed that stimuli have to be applied selectively to the *contracted* muscle in the gait cycle, i.e., alternating to the quadriceps and hamstring musculature according to their function. However, participants ran outdoors, so the investigators could not control, when the interval stimulations were activated at which muscles and EMS cycles of 2 s seem to cover at least an entire gait cycle.

## Conclusion

The effect of wbEMS is often compared to high-intensity interval training. Due to the stimulation, the classical recruitment principle is bypassed and type II-fibers can be recruited early on. We demonstrated that wbEMS can be safely applied during running; however, no additional effects on physiological determinants of endurance performance were observed when adding wbEMS to high-intensity running. Future investigations are needed which investigate different stimulation settings (interval vs. concurrent) and its effects on running economy. Furthermore, it is unclear if this training regimen might be more effective in individuals with a compromised aerobic power (e.g., during rehabilitation or for elderly participants).

### Supplementary Information

**Additional file 1: Supplementary Figure.** Heart rate and lactate.

## Data Availability

The datasets generated and analysed during the current study are not publicly available due to individual privacy, but are available from the corresponding author on reasonable request.
