# Supplementary material for: Running with whole-body electromyostimulation improves physiological determinants of endurance performance – a randomized control trial
Source: BMC Sports Sci Med Rehabil. 2023 Oct 4;15:126. doi: 10.1186/s13102-023-00739-8 (PMC10548652; doi:10.1186/s13102-023-00739-8)
Supplement: Supplementary file 1 — Additional file 1: Supplementary Figure. Heart rate and lactate. [file 13102_2023_739_MOESM1_ESM.pdf]

Supplementary Figure: Heart rate and lactate

Values of heart rate and lactate are illustrated for a representative participant for measures taken before and after the wbEMS intervention.

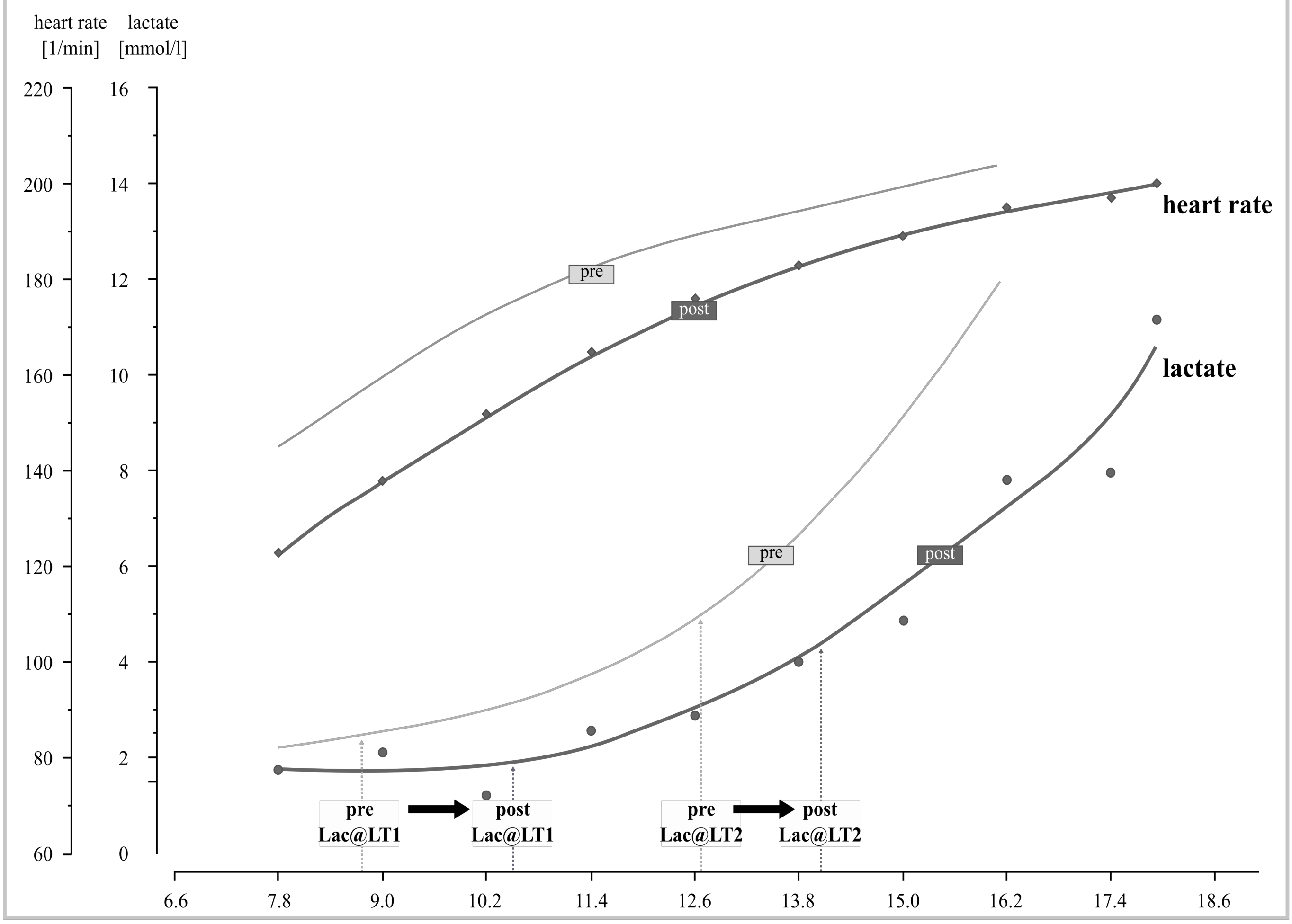

pre = baseline assessment (light grey), post = assessment following the 8-week intervention (dark grey), Lac = lactate, LT= lactate threshold, LT1 = aerobic lactate threshold, LT 2 = (indirect) anaerobic lactate threshold
